# High-resolution ultra-low field magnetic resonance imaging with a high-sensitivity sensing coil

**DOI:** 10.1063/5.0123692

**Published:** 2022-11-02

**Authors:** Igor Savukov, Young Jin Kim, Shaun Newman

**Affiliations:** MPA-Quantum, Los Alamos National Laboratory, P.O. Box 1663, MS-D454, Los Alamos, New Mexico 87545, USA

## Abstract

We present high-resolution magnetic resonance imaging (MRI) at ultra-low field (ULF) with a proton Larmor frequency of around 120 kHz. The key element is a specially designed high-sensitivity sensing coil in the shape of a solenoid with a few millimeter gap between windings to decrease the proximity effect and, hence, increase the coil’s quality ( Q) factor and sensitivity. External noise is strongly suppressed by enclosing the sensing coil in a copper cylindrical shield, large enough not to negatively affect the coil’s Q factor and sensitivity, measured to be 217 and 0.47 fT/Hz ${}^{1/2}$, respectively. To enhance small polarization of proton spins at ULF, a strong pulsed 0.1 T prepolarization field is applied, making the signal-to-noise ratio (SNR) of ULF MRI sufficient for high-quality imaging in a short time. We demonstrate ULF MRI of a copper sulfate solution phantom with a resolution of $1\times 1\times 8.5\;{\mathrm{mm}}^{3}$ and SNR of 10. The acquisition time is 6.3 min without averaging. The sensing coil size in the current realization can accommodate imaging objects of 9 cm in size, sufficient for hand, and it can be further increased for human head imaging in the future. Since the in-plane resolution of $1\times 1\;{\mathrm{mm}}^{2}$ is typical in anatomical medical imaging, this ULF MRI method can be an alternative low-cost, rapid, portable method for anatomical medical imaging of the human body or animals. This ULF MRI method can supplement other MRI methods, especially when such methods are restricted due to high cost, portability requirement, imaging artifacts, and other factors.

## I. INTRODUCTION

Ultra-low field (ULF) magnetic resonance imaging (MRI) using measurement magnetic fields from $10^{-6}$ to $10^{-3}$ T, corresponding to proton’s Larmor frequencies from 4.26 to 426 kHz (the gyromagnetic ratio 42.6 MHz/T), can supplement conventional several-Tesla MRI employed in medical practice. This is because ULF MRI systems, especially field-generation hardware, can be constructed of much lower cost and weight, compared to large and heavy permanent/superconducting magnets in conventional MRI systems, enabling portability. In addition, ULF MRI has many other advantages, for example, higher image contrast^1^ important for diagnosing anomalies, imaging in the presence of metal,^2^ and a possibility to be combined with other modalities, such as magneto-encephalograhpy (MEG).

ULF MRI was first introduced by researchers at UC Berkeley^3^ and later it was extensively investigated by the Los Alamos group^4–6^ and the Aalto University group.^7^ To partially compensate for the reduced signal-to-noise ratio (SNR) compared to conventional MRI, directly affecting the quality of ULF MRI, the ULF MRI methods featured prepolarization of the proton spins with a pulsed electromagnet and signal detection with sensitive low-temperature superconducting quantum interference device (SQUID) sensors reaching 1 fT/Hz ${}^{1/2}$ sensitivity. The Los Alamos group was the first to build and demonstrate combined SQUID-based ULF MRI and MEG in a single device,^5,8,9^ to realize one of the unique advantages of ULF MRI.

Unfortunately, SQUID-based ULF MRI systems are not practical for clinical applications because they require a bulky infrastructure of expensive liquid helium and a magnetically shielded room to suppress ambient magnetic field noise. These shortcomings can be overcome by replacing SQUIDs with a radio frequency (RF) optically pumped magnetometer (OPM)^10–12^ that can operate at ambient temperature in a simple and inexpensive magnetic shield and have sensitivity comparable to SQUIDs. The Savukov group at Los Alamos National Laboratory has demonstrated non-cryogenic anatomical ULF MRI of the human brain and hand based on RF OPMs with a resolution of a few mm.^13–15^ The Savukov group’s ULF MRI system additionally used a flux transformer, composed of two connected coils, combined with an RF OPM in order to make a sufficient separation between the RF OPM and MRI hardware, but effectively transfer the ULF MRI signal to the RF OPM. This unique configuration allowed us to minimize the negative effects of magnetic fields and gradients applied during ULF MRI measurements on the performance of the RF OPM. In addition, the Rosen group at Harvard University/Massachusetts General Hospital has demonstrated non-cryogenic anatomical ULF MRI of the human brain with a large room-temperature inductive coil^16^ without the prepolarization field but at higher Larmor frequencies. To improve the quality of images, the Rosen group used high-efficiency sampling strategies and fully refocused dynamic spin control. Besides, various coil-based magnetometers reaching a few tens of fT/Hz ${}^{1/2}$ were also designed for ULF nuclear magnetic resonance.^17–19^

In this paper, we introduce a practical ULF MRI approach based on a specially designed resonant sensing coil held at room temperature to sensitively detect the ULF MRI signal. Compared to the non-cryogenic ULF MRI systems mentioned above, the key benefit of this approach is to realize more compact and less expensive ULF MRI. The sensing coil in this approach is in the shape of a typical multi-layer solenoid, with a few mm gap between windings to reduce the proximity effect. This coil geometry effectively increases the quality ( Q) factor of the coil at ULF MRI frequencies. Increasing the Q factor is important because the magnetic sensitivity of the coil limited by the coil’s Johnson noise is expected to be improved by a factor of $\sqrt{Q}$. Owing to the use of the simple sensing coil, this ULF MRI approach can considerably reduce the cost and enable portability, valuable for clinical applications. Apart from the special sensing coil design giving theoretical sensitivity based on Q-factor measurements of 0.43 fT/Hz ${}^{1/2}$ at ∼100 kHz, the use of a completely closed copper shield and a capacitive voltage divider to reduce the sensing coil’s output impedance are essential to preserve the ultimate coil sensitivity in the realistic magnetic field environment. We demonstrate the use of this ULF MRI prototype to image a copper sulfate solution phantom with an in-plane resolution of $1\times 1\;{\mathrm{mm}}^{2}$ and an SNR of 10 in the acquisition time of 6.3 min without averaging, which exceeds significantly the resolution of our previous ULF MRI experiments at 120 kHz frequency.^13–15^

## II. METHOD

### A. Sensing coil

The magnetic sensitivity of a Q-factor resonant sensing coil is determined by the magnetic Johnson noise of the coil written as

$$\delta V_{J}=\sqrt{4k_{B}T\omega L/Q},$$ (1)

where $k_{B}$ is Boltzmann’s constant, T is the absolute temperature of the coil, ω is the operating angular frequency, and L is the inductance of the coil. In this work, our target operating ULF MRI frequency is around 100 kHz. Through Faraday’s law, the coil sensitivity is given by

$$\delta B=\frac{\sqrt{4k_{B}T\omega L/Q}}{\omega AN}=\frac{\sqrt{4k_{B}TL}}{AN\sqrt{\omega Q}},$$ (2)

where A and N are the cross-sectional area and the number of turns of the coil, respectively. According to Eq. (2), the coil sensitivity improves with increasing the Q factor, assuming all other parameters remain the same. Since $Q=\omega L/R_{ac}$ with $R_{ac}$ being the ac resistance of the coil that usually increases with frequency due to the skin and proximity effects,^20^ $R_{ac}$ should be reduced closer to the dc resistance value to avoid loss in Q. The skin effect prevents the RF current flowing through the total cross-sectional area of the wire, increasing the resistance of the coil. The skin effect can be suppressed by making the coil with the Litz wire of multiple strands of thin wire. The optimal size of the thin wire is dependent on the skin depth of the wire material and, hence, the observation frequency. On the other hand, the proximity effect in a multi-turn coil causes the RF currents to flow in neighboring wires and their dissipation,^21^ leading to the additional increase in the resistance of the coil. The proximity effect is strongly dependent on the coil geometry (distances between wires), hence optimizing the coil geometry is important. There are also losses associated with electrical fields in the wire material and self-capacitance of the coil, but in our case, these can be neglected.

**FIG. 1.**
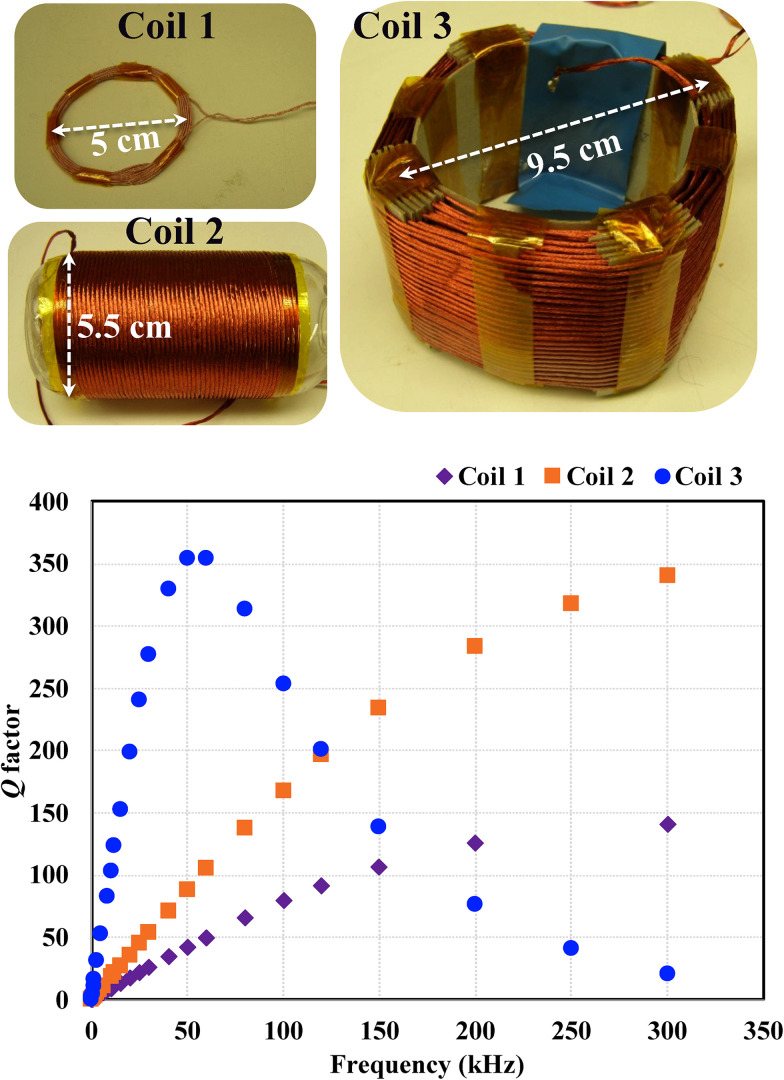
Measured Q factors of Coils 1–3. The Q factors of Coils 1 and 2 (a compact multi-turn circular coil and solenoid, respectively) gradually deviate from the linear dependence at frequencies above ∼50 kHz, with the maximum Q at a frequency exceeding 300 kHz. On the other hand, the Q factor of Coil 3 (a specialized multi-turn solenoid with extra spacing between windings) has its maximum around 100 kHz, optimal for ULF MRI measurements of this work.

We experimentally investigated an optimal coil geometry to minimize the proximity effect and, thus, maintain high Q at ∼100 kHz based on multiple coils, for example, Coils 1, 2, and 3 shown in Fig. 1. To be free from the contribution of the skin effect, these coils were made of the Litz wire of 25 strands of the 38 AWG wire for Coil 1 and 125 strands of 40 AWG wire for Coils 2 and 3. The Q factor of these coils was measured with a precision LQ meter (E4980AL-032) between 20 Hz and 300 kHz, which is the maximum frequency of the LQ meter. Coils 1 and 2 are a compact (no extra spacing) multi-turn circular coil and single-layer solenoid, respectively. Due to the proximity effect between neighboring windings, the measured Q factor of Coils 1 and 2 does not follow the linear dependence on the frequency, $Q=\omega L/R_{ac}$, if $R_{ac}$ were constant (Fig. 1). Furthermore, it is expected that these Q factors will reach their maximum values at a frequency larger than 300 kHz, away from the target ULF MRI frequencies.

**FIG. 2.**
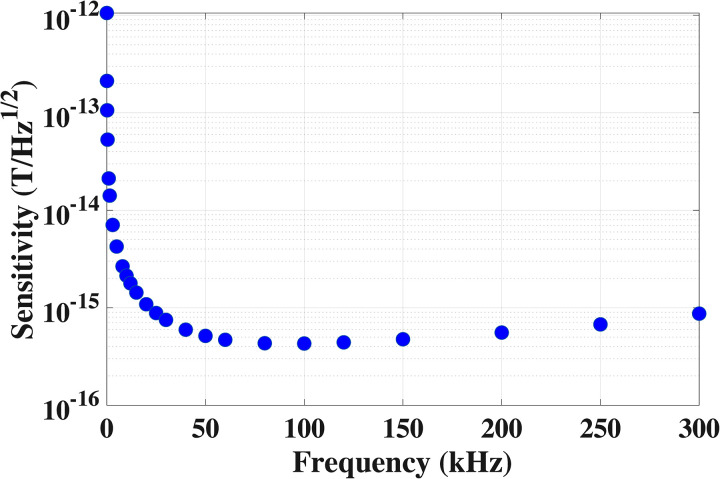
Theoretically estimated magnetic field sensitivity of Coil 3 based on the measured Q factor and Eq. (2) as a function of the frequency.

On the other hand, Coil 3 is a non-compact multi-turn solenoid (25 turns and eight layers with an average diameter of 9.5 cm and a height of 8.6 cm) with each winding separated by ∼2 mm in all directions to reduce the proximity effect. This wire spacing was chosen as a compromise between the need to increase the number of turns of the coil (thus, decrease the wire spacing) and reduce the proximity effect (thus, increase the wire spacing) in order to improve the magnetic field sensitivity of the coil. In this work, we did not ultimately optimize the wire spacing; instead, we started with the reasonably guessed 2 mm optimal wire spacing and observed that the near-optimal sensitivity was achieved in a relatively broad frequency range (Fig. 2), including our frequency of interest, 120 kHz, where ambient noise was relatively low. Unlike Coils 1 and 2, the measured Q factor of Coil 3 approaches its maximum value near 100 kHz, more suitable for this work. We expect that adjusting the winding separation can shift the maximum Q to different frequencies. This specialized multi-turn solenoid geometry improves the Q factor at the target ULF MRI frequencies around 100 kHz and, thus, can enhance the SNR of ULF MRI. To this end, we employed Coil 3 as a sensing coil in this work. The inductance of this coil was measured to be 3.65 mH. We theoretically estimated the magnetic field sensitivity of this coil based on Eq. (2) and the measured Q factor. The theoretical sensitivity as a function of the frequency is shown in Fig. 2. The coil can reach high sensitivity of 0.43 fT/Hz ${}^{1/2}$ around 100 kHz.

We experimentally measured the self-resonance frequencies of the three coils. In the case of Coil 3, using the LQ meter, we measured the absolute value of the coil’s impedance that can be written as $\left|Z|=1/|i\omega C+{\left(R_{ac}+i\omega L\right)}^{-1}\right|$ with C being the coil’s self-capacitance. The absolute value of the impedance reaches the maximum at the self-resonance frequency. The measurement was fit to the above impedance relation and the fit gave the self-capacitance of 97.5 pF and the self-resonance frequency of 269 kHz. In the case of Coils 1 and 2, their impedance did not reach the maximum within the operating frequency range of the LQ meter. Hence, we used a less accurate method in which voltage signals at different frequencies were applied to the coils and the responses of the coils were measured. We observed that the responses of Coils 1 and 2 were the largest at 2.1 and 1.8 MHz, respectively, which indicate the self-resonance frequencies of Coils 1 and 2.

### B. ULF MRI system

**FIG. 3.**
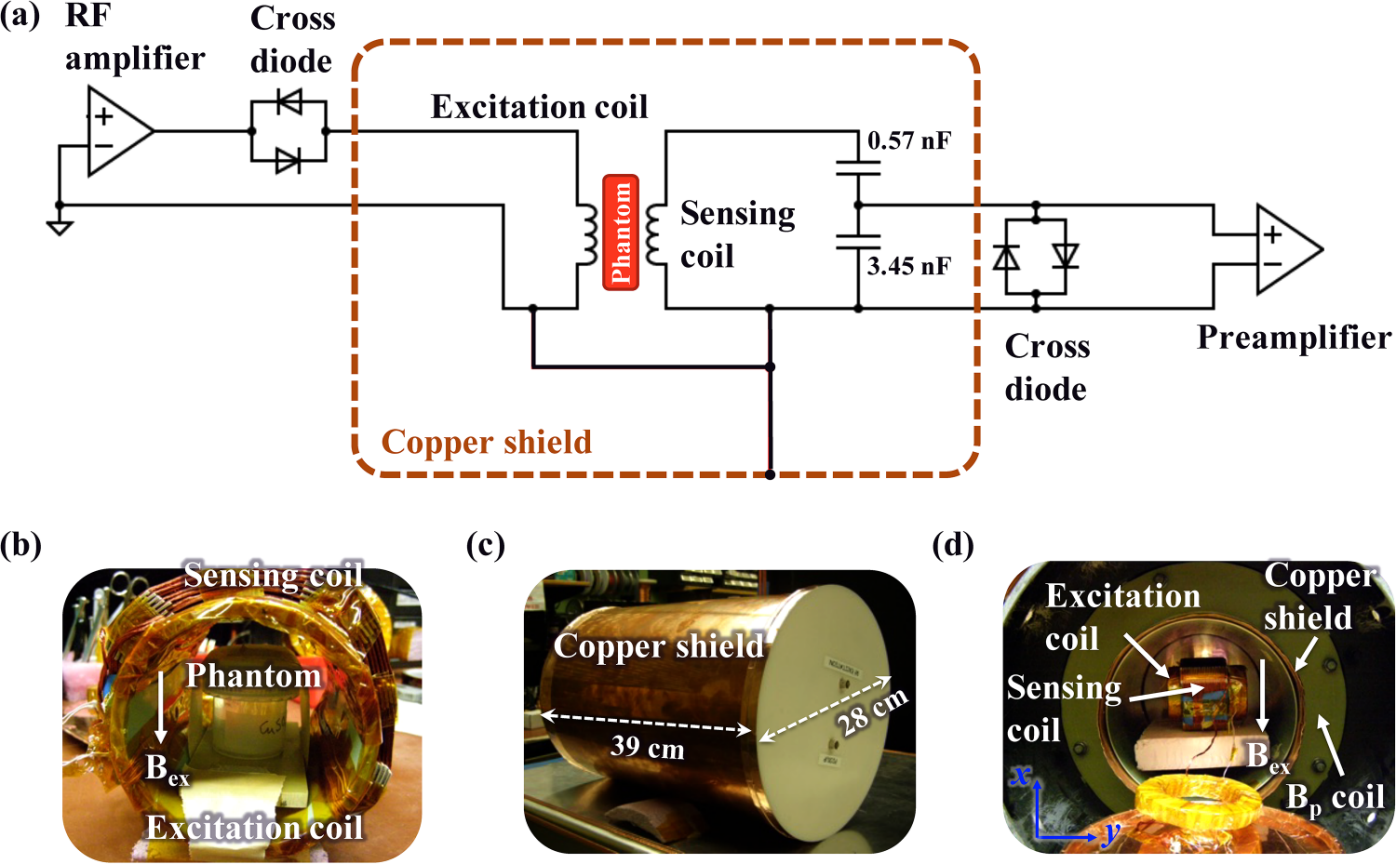
(a) A circuit diagram of a prototype ULF MRI system that contains an RF excitation system with an excitation saddle coil and a detection system based on the specially designed sensing coil (Coil 3 in Fig. 1). (b) Photograph of an assembly of the excitation and sensing coils. The excitation coil is inserted into the sensing coil by approximately aligning its center axes. A copper sulfate solution phantom is located at the center of these coils. (c) Photograph of a completely closed copper shield that houses the assembly of the coils and the copper sulfate solution phantom. (d) Photograph of the copper shield (the shield lid is opened only for showing its inner area) that located inside the bore of a MRI coil system composed of a prepolarization $B_{p}$ coil, a measurement $B_{m}$ coil (not shown), and gradient field coils (not shown).

We constructed a prototype ULF MRI system, as illustrated in Fig. 3. It comprises an RF excitation coil connected to a 16-bit analog output device (National Instruments PCI-6733) through an RF amplifier and the sensing coil (Coil 3 in Fig. 1) connected to a 24-bit data acquisition system (DAQ; National Instruments PCI-4472) through a preamplifier (Stanford Research Systems SR560). The cross diodes after the RF amplifier are added to reduce noise generated by the analog output device, while the cross diodes before the preamplifier are added to prevent possible large transient signals exceeding 5 V entering the preamplifier.^22^ The excitation and sensing coils were shielded from an ambient RF magnetic field by a completely closed copper cylindrical shield (28 cm diameter and 39 cm length) made of thin 1-mm copper sheet, shown in Fig. 3(c). To avoid ground loop decreasing the coil sensitivity, the excitation and sensing coil circuits as well as the copper shield were connected to the same grounding point.

As indicated in Fig. 3(a), the sensing coil is connected to a capacitive voltage divider to resonantly tune the sensing coil circuit to a desired ULF MRI frequency as well as to reduce the sensing coil’s output impedance. Unlike the coil design for high frequencies larger than MHz,^23^ it is not necessary to match the sensing coil circuit to the 50 Ω impedance at frequencies of ∼100 kHz because the size of the circuit and the lengths of the cables are much smaller than the signal wavelength of ∼3 km and, thus, the signal does not fluctuate due to interference effects in the cable. Although the sensing coil is located inside the copper shield, the coil is still connected to external cables and devices, resulting in additional noise in the detection system. The capacitive voltage divider, 0.57 and 3.45 nF capacitors connected in series, reduces the coil’s output impedance, making the detection system less sensitive to external noise. The divider, however, reduces the signal and has to be optimized so that the preamplifier noise of 4 nV/Hz ${}^{1/2}$ does not exceed the resonantly amplified coil Johnson noise after the divider of 13.8 nV/Hz ${}^{1/2}$. The ultimate noise of the detection system can be improved by using a more sensitive preamplifier or an OPM.

The excitation coil has a standard saddle coil geometry and was inserted into the sensing coil as shown in Figs. 3(b) and 3(d). Their center axes were approximately aligned. A copper sulfate solution phantom with relaxation times of $T_{1}=T_{2}\approx 0.2$ s was positioned in the center of the coil assembly. The excitation magnetic field, $B_{\mathrm{ex}}$, in the x direction as defined in Fig. 3(d), is perpendicular to the sensitive y direction of the sensing coil. This configuration minimizes the noise coming from the excitation coil. The assembly of the coils and the phantom were positioned in the center of the copper shield, as shown in Fig. 3(d).

In ULF MRI experiments, we used our home-built MRI coil system, which is composed of a four-coil Whiting–Lee $B_{m}$ coil generating a uniform measurement magnetic field, a prepolarization $B_{\;p}$ coil system (four short solenoid coils) increasing proton polarization, and a gradient coil system generating gradient fields of $G_{y}=dB_{z}/dy$ for frequency encoding and $G_{z}=dB_{z}/dz$ and $G_{x}=dB_{z}/dx$ for phase encoding. $G_{x}$ and $G_{y}$ gradients were generated by two orthogonal Golay coils, while the $G_{z}$ gradient was generated by a Maxwell pair. The fields of $B_{m}$ and $B_{\;p}$ are both in the z direction, constructed previously for a large-bore human head ULF MRI experiments.^14^ The copper shield was inserted inside the bore of the MRI coil system, as shown in Fig. 3(d).

**FIG. 4.**
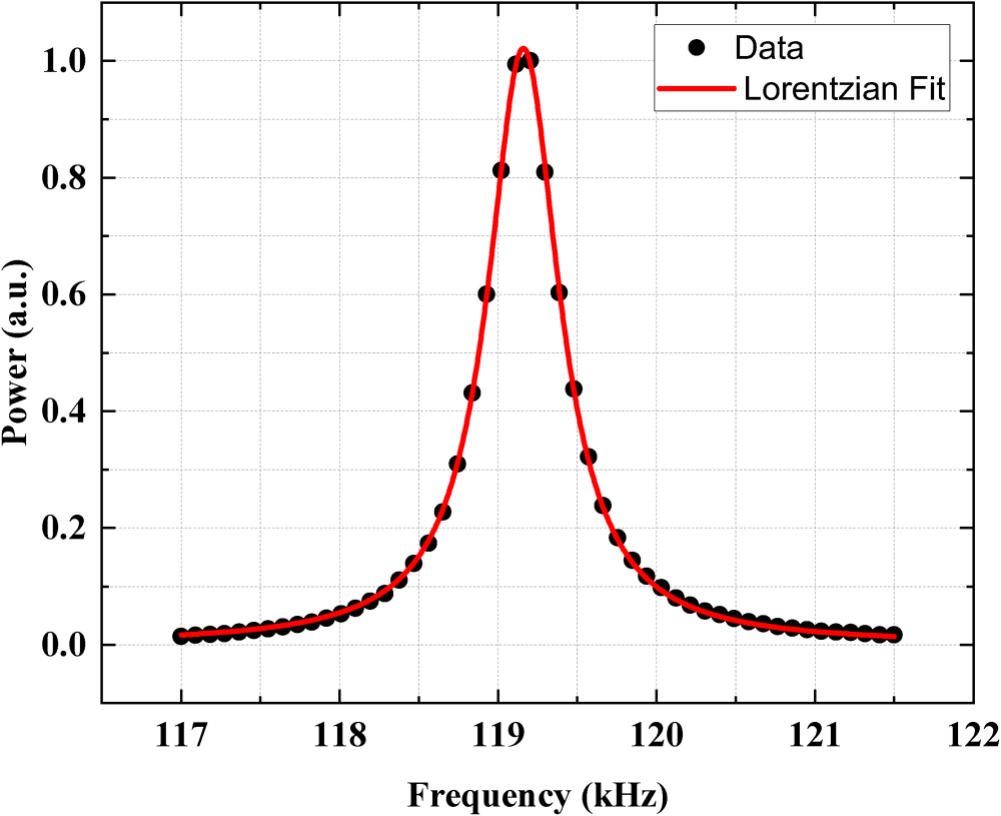
The resonance frequency of the ULF MRI system. The frequency response of the system was investigated by applying a sinusoidally varying magnetic field at different frequencies from 117 to 121.5 kHz to the excitation coil. The solid red line shows a Lorentzian fit with the bandwidth of 550 Hz and the resonance frequency of 119.16 kHz.

The resonance frequency of the ULF MRI detection system was measured to be 119.16 kHz. For this measurement, a sine wave of sufficiently small amplitude, not to saturate the amplifier, was applied to the excitation coil and the peak at the excitation frequency observed in the spectrum obtained with the fast Fourier transform (FFT) was measured with the sensing coil. The measured power spectrum is shown in Fig. 4. The data were fit to a Lorentzian function, $f(\nu )=a_{0}+a_{1}/[4{\left(\nu -\nu_{0}\right)}^{2}+\Delta \nu^{2}]$, where ν is the frequency of the applied sine wave. The fit gives the bandwidth of Δν=550 Hz, which corresponds to Q=217 through the relation of $Q=\nu_{0}/\Delta \nu$. The measured Q factor matches well the value at 120 kHz in Fig. 1, indicating that the Q factor of the sensing coil was not reduced due to the copper shield, the excitation coil, and electrical connections. However, when we used a smaller, thicker copper shield (16 cm diameter, 28 cm height, and 3 mm thickness) that was closer to the sensing coil, we observed that the Q factor reached only 120 due to eddy-current losses in the shield. Such losses would increase the coil noise by a factor of 1.34.

The sensing coil’s Johnson noise (before the divider) is estimated to be 0.45 nV/Hz ${}^{1/2}$ at 119.16 kHz based on Eq. (1) and experimental values of the sensing coil, Q=217 and L=3.65 mH. The measured coil’s noise was at 0.49 nV/Hz ${}^{1/2}$, which agrees with the estimated value. Through Eq. (2), the measured magnetic field sensitivity of the sensing coil is 0.47 fT/Hz ${}^{1/2}$. We also calibrated the coil sensitivity by applying a known magnetic field and the measured response of the coil agreed with the theoretical response for the frequency range up to 180 kHz below the self-resonance frequency. The 0.47 fT/Hz ${}^{1/2}$ sensitivity is about two times better than those achieved with ULF MRI systems based on SQUIDs^4–6^ and RF OPMs.^15^ SNR-wise, the two times sensitivity improvement is four times imaging time acceleration to reach the same SNR.

### C. ULF MRI procedure

**FIG. 5.**
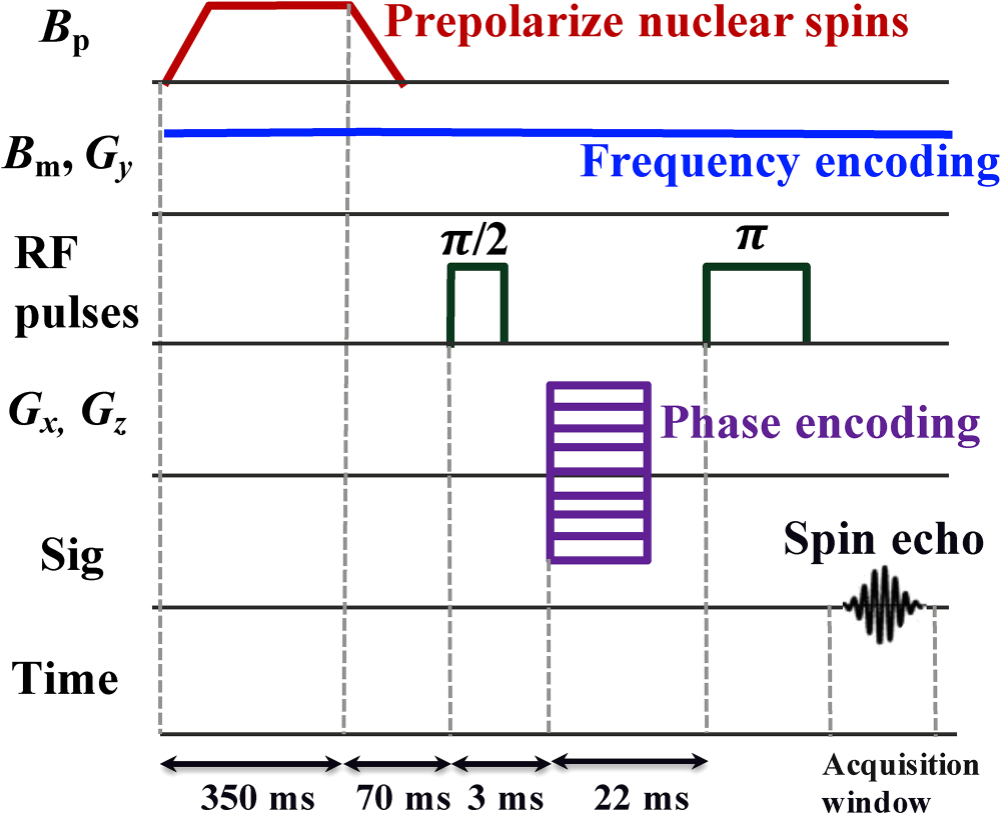
3D ULF MRI procedure with one frequency encoding and two phase encoding gradients. The measurement $B_{m}$ and frequency encoding $G_{y}$ fields are always on during the measurements. A pulsed prepolarization $B_{p}$ field for 350 ms followed by spin-echo pulses ( π/2- and π-pulses) is applied to the copper sulfate solution phantom. Phase encoding $G_{x}$ and $G_{z}$ fields are applied between π/2 and π pulses. The data recording starts at 350 ms and 3D imaging is realized with data inside the acquisition window. One phase encoding cycle takes 600 ms.

Figure 5 shows the imaging sequence that we applied for a 3D ULF MRI. The pulses of the sequence and data recording were controlled by a home-built LabVIEW program. Initially, the pulsed prepolarization field, $B_{p}=100$ mT, was applied to the copper sulfate solution phantom for 350 ms. The measurement field, $B_{m}=2.8$ mT collinear with $B_{p}$, sets the MRI frequency to 119.16 kHz. The frequency encoding gradient field $G_{y}$ was turned on during the measurements. A π/2-pulse with a duration of 0.25 ms was applied at 70 ms after turning off the $B_{p}$ in order to have a sufficient time for $B_{p}$ to ramp down not to affect the nuclear precession around $B_{m}$. Then, gradients, $G_{x}$ and $G_{z}$, with a duration of 20 ms were applied at 3 ms after the π/2-pulse to generate spatially phase-encoded precession signals coming from different areas of the imaging phantom. At 25 ms after the π/2-pulse, a π-pulse of a duration of 0.5 ms was applied to reverse the phase evolution of spins and remove the effects of external gradients and gradients due to the $B_{m}$ coil imperfections. The signal was recorded after the $B_{p}$ was turned off, and its part after the spin-echo pulses inside the acquisition window was used for imaging. The start time and the duration of the acquisition window were adjusted to optimize SNR and resolution.

The data were amplified by the SRS preamplifier with the bandpass filter set to 10–300 kHz and then down-converted to low frequency around 2 kHz with an analog mixer (Mini-Circuits ZAD-8). The down-converted signals were acquired by the 24-bit DAQ with a 10 kHz sampling rate (thus reducing the data burden to the DAQ computer) and digitally filtered by a 4th-order software filter in the range between 1.2 and 3 kHz to facilitate the observation of the spin-echo signals in the time domain. This filter did not distort significantly the image intensities.

One phase encoding cycle took 600 ms, leading to the total acquisition time of 6.3 min with 71 and nine phase encoding steps in the horizontal ( z) and depth ( x) directions, respectively. These phase encoding steps were chosen for the ULF MRI measurements described in Sec. III.

## III. EXPERIMENTAL RESULTS AND DISCUSSION

**FIG. 6.**
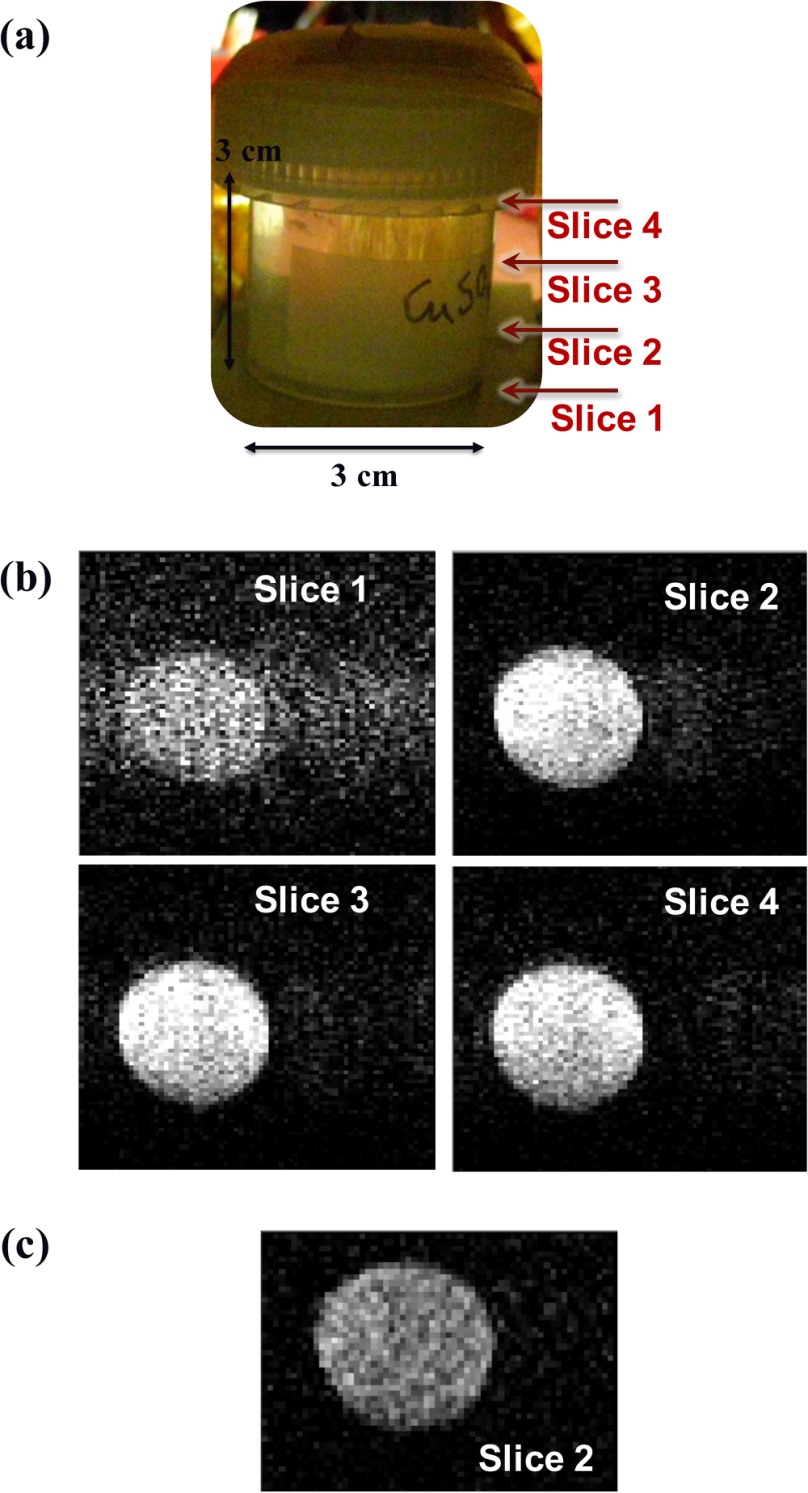
(a) Photograph of the copper sulfate solution phantom showing approximate positions of each imaging slice with slice thickness 8.5 mm. (b) Phantom images of each slice with $1\times 1\;{\mathrm{mm}}^{2}$ in-plane resolution. (c) A phantom image of slice 2 when using the smaller, thicker copper shield, resulting in the Q factor of the sensing coil of 120 and significant SNR reduction.

We performed ULF MRI measurements with the ULF MRI procedure. Figure 6(b) shows ULF MRI of four slices of the copper sulfate solution phantom (3 cm diameter and 3 cm height), each approximate slice position indicated in Fig. 6(a), in a single scan without data averaging. SNR of slice 1 is worse than the others because the bottom of the phantom was partially covered. We have achieved a high resolution of $1\times 1\times 8.5\;{\mathrm{mm}}^{3}$ and a high SNR of 10 in the total acquisition time of 6.3 min in a single scan without averaging. On the other hand, Fig. 6(c) shows single-scanned ULF MRI of slice 2 of the copper sulfate solution phantom obtained using the smaller, thicker copper shield that spoiled the Q factor of the sensing coil to 120. Its in-plane resolution reached also $1\times 1\;{\mathrm{mm}}^{2}$; however, the total acquisition time was 9.4 min, and the SNR was much smaller. Therefore, preserving the Q factor of the sensing coil in the ULF MRI system is important to obtain high quality images.

Since the $1\times 1\;{\mathrm{mm}}^{2}$ in-plane resolution is sufficient for anatomical medical imaging, this ULF MRI method can be applied for low-cost, rapid high-quality imaging of the human body and head or animals to accelerate a diagnosis of diseases. The simple sensing coil can potentially allow us to develop a portable MRI, valuable for clinical applications in hospitals. This ULF MRI method can supplement other MRI methods, especially when such methods are restricted due to high cost, space availability, portability requirement, imaging artifacts, patient’s comfort, and other factors.

A combination of MRI and MEG in the same instrument is one example in which ULF MRI can uniquely be used, while the combination of MEG even with low-field MRI systems using measurement magnetic fields 0.1 T is impossible.

## IV. CONCLUSION

We performed ULF MRI measurements using the prototype ULF MRI system based on the specially designed sensing coil with a high magnetic field sensitivity of 0.47 fT/Hz ${}^{1/2}$ around 120 kHz, the capacitive voltage divider, and the completely closed copper shield. We obtained ULF MRI of the copper sulfate solution phantom with a high resolution of $1\times 1\times 8.5\;{\mathrm{mm}}^{3}$ and a high SNR of 10 in the total acquisition time of 6.3 min in a single scan without averaging. This ULF MRI system can be applied to small animal imaging without modifications but can be further developed for human subjects.

## Data Availability

The data that support the findings of this study are available from the corresponding author upon reasonable request.

## References

[1] R. Kraus, M. Espy, P. Magnelind, and P. Volegov, *Ultra-Low Field Nuclear Magnetic Resonance: A New MRI Regime* (Oxford University Press, 2014).

[2] R. D. Venook, N. I. Matter, M. Ramachandran, S. E. Ungersma, G. E. Gold, N. J. Giori, A. Macovski, G. C. Scott, and S. M. Conolly, Magn. Reson. Med. 56, 177 (2006). 10.1002/mrm.20927 16724303

[3] R. McDermott, S. Lee, B. ten Haken, A. H. Trabesinger, A. Pines, and J. Clarke, Proc. Natl. Acad. Sci. U.S.A. 101, 7857 (2004). 10.1073/pnas.0402382101 15141077 PMC419521

[4] V. S. Zotev, P. L. Volegov, A. N. Matlashov, M. A. Espy, J. C. Mosher, and R. H. Kraus, J. Magn. Reson. 192, 197 (2008). 10.1016/j.jmr.2008.02.015 18328753 PMC2483697

[5] V. S. Zotev, A. N. Matlashov, P. L. Volegov, I. M. Savukov, M. A. Espy, J. C. Mosher, J. J. Gomez, and R. H. Kraus, J. Magn. Reson. 194, 115 (2008). 10.1016/j.jmr.2008.06.007 18619876 PMC2556894

[6] M. Espy, A. Matlashov, and P. Volegov, J. Magn. Reson. 229, 127 (2013). 10.1016/j.jmr.2013.02.009 23452838

[7] P. T. Vesanen, J. O. Nieminen, K. C. J. Zevenhoven, J. Dabek, L. T. Parkkonen, A. V. Zhdanov, J. Luomahaara, J. Hassel, J. Penttilä, J. Simola, A. I. Ahonen, J. P. Mäkelä, and R. J. Ilmoniemi, Magn. Reson. Med. 69, 1795 (2013). 10.1002/mrm.24413 22807201

[8] V. S. Zotev, A. N. Matlachov, P. L. Volegov, H. J. Sandin, M. A. Espy, J. C. Mosher, A. V. Urbaitis, S. G. Newman, and R. H. Kraus, IEEE Trans. Appl. Supercond. 17, 839 (2007). 10.1109/TASC.2007.898198

[9] A. Matlashov, E. Burmistrov, P. Magnelind, L. Schultz, A. Urbaitis, P. Volegov, J. Yoder, and M. Espy, Phys. C 482, 19 (2012). 10.1016/j.physc.2012.04.028

[10] I. Savukov and M. G. Boshier, Sensors 16, 1691 (2016). 10.3390/s16101691 PMC5087479 27754358

[11] I. M. Savukov, S. J. Seltzer, M. V. Romalis, and K. L. Sauer, Phys. Rev. Lett. 95, 063004 (2005). 10.1103/PhysRevLett.95.063004 16090946

[12] S.-K. Lee, K. L. Sauer, S. J. Seltzer, O. Alem, and M. V. Romalis, Appl. Phys. Lett. 89, 214106 (2006). 10.1063/1.2390643

[13] I. Savukov, T. Karaulanov, A. Castro, P. Volegov, A. Matlashov, A. Urbatis, J. Gomez, and M. Espy, J. Magn. Reson. 211, 101 (2011). 10.1016/j.jmr.2011.05.011 21700482 PMC3143263

[14] I. Savukov and T. Karaulanov, Appl. Phys. Lett. 103, 043703 (2013). 10.1063/1.4816433 PMC3739803 23964134

[15] I. Savukov and T. Karaulanov, J. Magn. Reson. 231, 39 (2013). 10.1016/j.jmr.2013.02.020 23567881

[16] M. Sarracanie, C. D. LaPierre, N. Salameh, D. E. J. Waddington, T. Witzel, and M. S. Rosen, Sci. Rep. 5, 15177 (2015). 10.1038/srep15177 26469756 PMC4606787

[17] R. Pellicer-Guridi, M. W. Vogel, D. C. Reutens, and V. Vegh, Sci. Rep. 7, 2269 (2017). 10.1038/s41598-017-02099-z 28536460 PMC5442107

[18] A. N. Matlashov, L. J. Schultz, M. A. Espy, R. H. Kraus, I. M. Savukov, P. L. Volegov, and C. J. Wurden, IEEE Trans. Appl. Supercond. 21, 465 (2011). 10.1109/TASC.2010.2089402 21747638 PMC3131692

[19] S. Tumanski, Meas. Sci. Technol. 18, R31 (2007). 10.1088/0957-0233/18/3/R01

[20] I. Savukov, S. Seltzer, and M. Romalis, J. Magn. Reson. 185, 214 (2007). 10.1016/j.jmr.2006.12.012 17208476

[21] X. Nan and C. Sullivan, in *IEEE 34th Annual Conference on Power Electronics Specialist, 2003. PESC ’03.* (IEEE, 2003), Vol. 2, pp. 853–860.

[22] I. Savukov, Y. J. Kim, and G. Schultz, J. Magn. Reson. 317, 106780 (2020). 10.1016/j.jmr.2020.106780 32688163

[23] D. Hoult and R. Richards, J. Magn. Reson. (1969) 24, 71 (1976). 10.1016/0022-2364(76)90233-X 22152352

